# Modeling Sage data with a truncated gamma-Poisson model

**DOI:** 10.1186/1471-2105-7-157

**Published:** 2006-03-20

**Authors:** Helene H Thygesen, Aeilko H Zwinderman

**Affiliations:** 1 Clinical Epidemiology and Biostatistics, Academisch Medisch Centrum, University of Amsterdam, Meibergdreef 9, 1100 DD Amsterdam, The Netherlands

## Abstract

**Background:**

Serial Analysis of Gene Expressions (SAGE) produces gene expression measurements on a discrete scale, due to the finite number of molecules in the sample. This means that part of the variance in SAGE data should be understood as the sampling error in a binomial or Poisson distribution, whereas other variance sources, in particular biological variance, should be modeled using a continuous distribution function, i.e. a prior on the intensity of the Poisson distribution. One challenge is that such a model predicts a large number of genes with zero counts, which cannot be observed.

**Results:**

We present a hierarchical Poisson model with a gamma prior and three different algorithms for estimating the parameters in the model. It turns out that the rate parameter in the gamma distribution can be estimated on the basis of a single SAGE library, whereas the estimate of the shape parameter becomes unstable. This means that the number of zero counts cannot be estimated reliably. When a bivariate model is applied to two SAGE libraries, however, the number of predicted zero counts becomes more stable and in approximate agreement with the number of transcripts observed across a large number of experiments. In all the libraries we analyzed there was a small population of very highly expressed tags, typically 1% of the tags, that could not be accounted for by the model. To handle those tags we chose to augment our model with a non-parametric component. We also show some results based on a log-normal distribution instead of the gamma distribution.

**Conclusion:**

By modeling SAGE data with a hierarchical Poisson model it is possible to separate the sampling variance from the variance in gene expression. If expression levels are reported at the gene level rather than at the tag level, genes mapped to multiple tags must be kept separate, since their expression levels show a different statistical behavior. A log-normal prior provided a better fit to our data than the gamma prior, but except for a small subpopulation of tags with very high counts, the two priors are similar.

## Background

In Serial Analysis of Gene Expression (SAGE), mRNA is extracted from a tissue sample and converted to cDNA, from which oligonucleotides (so-called SAGE tags) at specific locations in the cDNA fragments are extracted and amplified using PCR. Those tags are either ten or seventeen bases long, depending on the experimental protocol. Sequencing the PCR product, it is possible to establish the number of copies of each tag extracted. (For an elaborate description of the technology, see Velculescu [1]). Ideally, there would be a one-to-one relation between tags and genes, so that the number of copies of a tag would be an indicator of the rate of transcription of the corresponding gene. Suppose the tissue sample contained *n*_*t *_copies of tag *t *each of which have a probability *p *of being extracted. The exact magnitude of *p *is unknown (and depends on experimental circumstances) but is certainly much smaller than 1 (Kuznetsov [2])), which suggests modeling the number *y*_*t *_of observed copies of tag *t *(the so-called SAGE count) as Poisson distributed with intensity *λ*_*t *_= *pn*_*t*_.

A Poisson model predicts a (large) number of zero counts, i.e. tags with positive *lambda-values *that just happened not to be counted. Those cannot be distinguished from tags that do not exist at all or are never transcribed. The problem of estimating the total number of expressible tags (the size of the transcriptome) was studied by Stern [3], who found the number of tags expressed at each level to be inversely proportional to the square of the expression level. Stern concluded that the size of the transcriptome could not be reliably estimated from SAGE data. Part of the problem is that a substantial part of the low-expressed tags may be artifactual, which is difficult to incorporate in the model. (Some authors have developed statistical models for SAGE data that take artifactually low counts into account, see Blades [4], Beissbarth [5] and Anisomov [6]). Kuznetsov [7], [8] modeled the SAGE data using a discrete Pareto-like distribution and found that his model was able to predict the number of transcripts expressed at a level of ≥ 1 copy per cell. Although this was a major breakthrough, the discrete Pareto-like distribution models the counts directly, which means that sample variance is not explicitly separated from the variance in gene expression. The model that we explore in this paper is an hierarchical Poisson model, i.e. a Poisson distribution with some prior distribution *f *of Poisson parameter *λ*

*Y*_*t *_~ *Poisson*(*λ*_*t*_), *λ*_*t *_~ *f*(·,*θ*)     (1)

where *Y*_*t *_is the observed count for tag *t*, *λ*_*t *_is the "true" expression level of tag *t *and *θ *is some parameter in the model. For the prior *f *we tried a number of candidates (gamma, mixture of two gamma's, log-normal, Pareto). The gamma prior turned out to provide a good fit to the distributions of the tag counts for counts lower than a certain threshold, typically the 98th or 99th percentile. Attempts to model the tag counts above that threshold with a second gamma-distributed component failed, not surprisingly since the number of tags in that range was too small to support meaningful estimates.

For the purpose of this paper we choose the gamma distribution, whose parameters were estimated with an empirical Bayesian approach [9]. The choice of the Gamma distribution was motivated by mathematical convenience only. The Gamma distribution is the conjugate prior of the Poisson distribution, i.e. if the parameters *α *and *β *are known, the posterior distribution of *λ*_*t *_given *y*_*t *_is distributed as Gamma(*α *+ *y*_*t*_, *β *+ 1) This is convenient because the posterior distribution of *γ *represents our knowledge of the true gene expression after the SAGE count has been observed. Also, since 1/*β *is a scale parameter in the Gamma distribution, libraries of different size can be compared. Other things being equal, we expect the estimated value of *β *to be inversely proportional to the library size.

The marginal distribution of *Y *becomes a negative binomial distribution:

P(Y=y)=Γ(y+α)Γ(α)y!βα(β+1)y+α     (2)
 MathType@MTEF@5@5@+=feaafiart1ev1aaatCvAUfKttLearuWrP9MDH5MBPbIqV92AaeXatLxBI9gBaebbnrfifHhDYfgasaacH8akY=wiFfYdH8Gipec8Eeeu0xXdbba9frFj0=OqFfea0dXdd9vqai=hGuQ8kuc9pgc9s8qqaq=dirpe0xb9q8qiLsFr0=vr0=vr0dc8meaabaqaciaacaGaaeqabaqabeGadaaakeaacqWGqbaucqGGOaakcqWGzbqwcqGH9aqpcqWG5bqEcqGGPaqkcqGH9aqpdaWcaaqaaiabfo5ahjabcIcaOiabdMha5jabgUcaRGGaciab=f7aHjabcMcaPaqaaiabfo5ahjabcIcaOiab=f7aHjabcMcaPiabdMha5jabcgcaHaaadaWcaaqaaiab=j7aInaaCaaaleqabaGae8xSdegaaaGcbaGaeiikaGIae8NSdiMaey4kaSIaeGymaeJaeiykaKYaaWbaaSqabeaacqWG5bqEcqGHRaWkcqWFXoqyaaaaaOGaaCzcaiaaxMaadaqadaqaaiabikdaYaGaayjkaiaawMcaaaaa@52F9@

and in particular

P(Y=0)=(ββ+1)α     (3)
 MathType@MTEF@5@5@+=feaafiart1ev1aaatCvAUfKttLearuWrP9MDH5MBPbIqV92AaeXatLxBI9gBaebbnrfifHhDYfgasaacH8akY=wiFfYdH8Gipec8Eeeu0xXdbba9frFj0=OqFfea0dXdd9vqai=hGuQ8kuc9pgc9s8qqaq=dirpe0xb9q8qiLsFr0=vr0=vr0dc8meaabaqaciaacaGaaeqabaqabeGadaaakeaacqWGqbaucqGGOaakcqWGzbqwcqGH9aqpcqaIWaamcqGGPaqkcqGH9aqpdaqadaqaamaalaaabaacciGae8NSdigabaGae8NSdiMaey4kaSIaeGymaedaaaGaayjkaiaawMcaamaaCaaaleqabaGae8xSdegaaOGaaCzcaiaaxMaadaqadaqaaiabiodaZaGaayjkaiaawMcaaaaa@3FFD@

Since the zero counts are not recorded, the counts of the recorded tags follow a zero-truncated negative binomial distribution

P(Y=y|Y>0)=Γ(y+α)Γ(α)y!βα(β+1)y+α11−(ββ+1)α     (4)
 MathType@MTEF@5@5@+=feaafiart1ev1aaatCvAUfKttLearuWrP9MDH5MBPbIqV92AaeXatLxBI9gBaebbnrfifHhDYfgasaacH8akY=wiFfYdH8Gipec8Eeeu0xXdbba9frFj0=OqFfea0dXdd9vqai=hGuQ8kuc9pgc9s8qqaq=dirpe0xb9q8qiLsFr0=vr0=vr0dc8meaabaqaciaacaGaaeqabaqabeGadaaakeaacqWGqbaucqGGOaakcqWGzbqwcqGH9aqpcqWG5bqEcqGG8baFcqWGzbqwcqGH+aGpcqaIWaamcqGGPaqkcqGH9aqpdaWcaaqaaiabfo5ahjabcIcaOiabdMha5jabgUcaRGGaciab=f7aHjabcMcaPaqaaiabfo5ahjabcIcaOiab=f7aHjabcMcaPiabdMha5jabcgcaHaaadaWcaaqaaiab=j7aInaaCaaaleqabaGae8xSdegaaaGcbaGaeiikaGIae8NSdiMaey4kaSIaeGymaeJaeiykaKYaaWbaaSqabeaacqWG5bqEcqGHRaWkcqWFXoqyaaaaaOWaaSaaaeaacqaIXaqmaeaacqaIXaqmcqGHsisldaqadaqaamaalaaabaGae8NSdigabaGae8NSdiMaey4kaSIaeGymaedaaaGaayjkaiaawMcaamaaCaaaleqabaGae8xSdegaaaaakiaaxMaacaWLjaWaaeWaaeaacqaI0aanaiaawIcacaGLPaaaaaa@62FF@

The zero-truncated negative binomial distribution has been studied by several authors, mainly for modeling group sizes. See Johnson [10] for an overview. Schenzle [11] studied the efficiency of various estimation methods for the parameters and reached the conclusion that for *α *< 1 heuristic methods do not work and ML estimation should be used instead.

If such a model can provide a good fit to the data, it will be useful for several purposes. For example, knowing the posterior distribution of *λ*_*t *_given *Y*_*t*_, one can construct a variance-stabilizing transform for a SAGE library. Also, in order to assess the sensitivity of the SAGE technology with respect to genes with low expression levels, one needs to know the distribution of *λ*. The idea of applying Poisson models to SAGE data is not new. Cai [12] found a Poisson-based gene clustering algorithm to work better than one based on Euclidian distances. And several authors (Vencio [13], Ruijter [14]) have compared Poisson-based tests for differentiation in gene expression between libraries. However, those tests always look at a single SAGE tag at a time and tests for difference in the parameter *λ *across the libraries. As noted by Baggerly [15], it is possible that a model that incorporates all tags simultaneously, analogous to the models applied to microarray data, would be more powerful. This motivates our attempt to model the behavior of *λ *across genes. In this paper, we present a truncated Gamma-Poisson model augmented with a non-parametric component, as well as a bivariate truncated Gamma-Poisson model that can be used to comparing two libraries, and we show how the parameters can be estimated in those models.

## Results

### Untruncated gamma-Poisson, ML and method of moments

In the human transcriptome map, genes with zero counts were reported, which allowed for the untruncated negative binomial model to be used. While the maximum likelihood (Johnson [10]) method worked quite well with simulated data, it did not provide meaningful results with the data from the Human Transcriptome Map, presumably because of the failure to account for a small population of tags with extremely high counts (typically 1% of all tags). Because those tags had high influence on the likelihood function, it was necessary to take them into account. Therefore, we assumed the expressed tags to be a mixture of two populations: a large population of tags with expression levels below some threshold *k*, described by the gamma-poisson model, and a small population of tags with expressions at or above *k*, described by a non-parametric distribution. Because the maximum likelihood estimator itself requires iteration, we decided to use a heuristic to choose the threshold *k *and the start-guess for the maximum likelihood estimator (See the Methods section).

**Figure 1:**
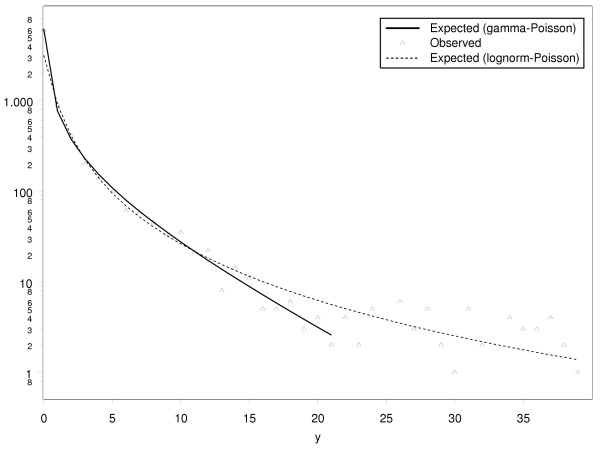
Frequency of SAGE counts (restricted to genes mapped to a single tag) of library N225.111k, versus the expected frequencies on the basis of the untruncated model with non-parametric component for high-expressed tags. Compared to the expected frequencies in the truncated lognorm-Poisson-model.

As for the Human Transcriptome Map, the model provided a good fit to the counts for tags of low to moderate expression, but for all 72 libraries it was necessary to assume a non-parametric component accounting for the extremely strongly expressed genes. Figure 1 shows the expected frequencies versus the observed frequencies for HTM library N225.111k (a neuroblastoma cell line), which was the largest library.

The quartiles of the estimated parameters for the 72 libraries are shown in table 1.

We expected the estimated parameter *β *to scale with the inverse of the library size, and indeed we found a clear negative correlation between log(*β*) and log(library size), R = -0.78.

Using the estimates from N225.111k (*α *= 0.146, *β *= 0.171) as an example, variance of *λ *is *α*/*β*^2 ^= 1.55.

The average sampling variance is identical to the mean, *α*/*β *= 0.27. Those average variances, however, hide huge differences in information content between tags with different expression levels: The coefficient of variation of the posterior *λ*_*t *_given a count *y*_*t *_is 1/yt+α
 MathType@MTEF@5@5@+=feaafiart1ev1aaatCvAUfKttLearuWrP9MDH5MBPbIqV92AaeXatLxBI9gBaebbnrfifHhDYfgasaacH8akY=wiFfYdH8Gipec8Eeeu0xXdbba9frFj0=OqFfea0dXdd9vqai=hGuQ8kuc9pgc9s8qqaq=dirpe0xb9q8qiLsFr0=vr0=vr0dc8meaabaqaciaacaGaaeqabaqabeGadaaakeaacqaIXaqmcqGGVaWldaGcaaqaaiabdMha5naaBaaaleaacqWG0baDaeqaaOGaey4kaSccciGae8xSdegaleqaaaaa@3447@

### Truncated gamma-Poisson, Maximum Likelihood and MCMC

The log-likelihood in the truncated negative binomial model, without the non-parametric component for the high-expressed tags, is

∑t(αlog(β)+∑j=0Yt−1log(α+j)−(α+Yt)log(β+1)−log(1−(β1+β)α))     (5)
 MathType@MTEF@5@5@+=feaafiart1ev1aaatCvAUfKttLearuWrP9MDH5MBPbIqV92AaeXatLxBI9gBaebbnrfifHhDYfgasaacH8akY=wiFfYdH8Gipec8Eeeu0xXdbba9frFj0=OqFfea0dXdd9vqai=hGuQ8kuc9pgc9s8qqaq=dirpe0xb9q8qiLsFr0=vr0=vr0dc8meaabaqaciaacaGaaeqabaqabeGadaaakeaadaaeqbqaamaabmaabaacciGae8xSde2exLMBbXgBcf2CPn2qVrwzqf2zLnharyGvLjhzH5wyaGabciaa+XgacaGFVbGaa43zaiabcIcaOiab=j7aIjabcMcaPiabgUcaRmaaqahabaGaa4hBaiaa+9gacaGFNbGaeiikaGIae8xSdeMaey4kaSIaemOAaOMaeiykaKIaeyOeI0IaeiikaGIae8xSdeMaey4kaSIaemywaK1aaSbaaSqaaiabdsha0bqabaGccqGGPaqkcaGFSbGaa43Baiaa+DgacqGGOaakcqWFYoGycqGHRaWkcqaIXaqmcqGGPaqkcqGHsislcaGFSbGaa43Baiaa+DgadaqadaqaaiabigdaXiabgkHiTmaabmaabaWaaSaaaeaacqWFYoGyaeaacqaIXaqmcqGHRaWkcqWFYoGyaaaacaGLOaGaayzkaaWaaWbaaSqabeaacqWFXoqyaaaakiaawIcacaGLPaaaaSqaaiabdQgaQjabg2da9iabicdaWaqaaiabdMfaznaaBaaameaacqWG0baDaeqaaSGaeyOeI0IaeGymaedaniabggHiLdaakiaawIcacaGLPaaaaSqaaiabdsha0bqab0GaeyyeIuoakiaaxMaacaWLjaWaaeWaaeaacqaI1aqnaiaawIcacaGLPaaaaaa@7AD0@

We augmented this model with a non-parametric component for the high-expressed genes in the same way as the untruncated model described above.

While the estimate of the rate parameter in the truncated model became reasonably stable, and, as expected, approximately inversely proportional to the library size, the shape parameter became unstable, in particular with real data but also with simulated data.

The rate parameter (*α*) in most libraries converged towards the lower bound specified in the call to the maximization routine (0.001). A curious exception was GSM1130, a small library with only 17004 tags, which gave an estimate of *α *of 0.77 in the censored model as opposed to 0.05 in the uncensored model. For all the other libraries, the estimate of *β *for the censored model was in agreement with that from the uncensored model (mean ratio = 1.06, SD= 0.10). Computations with simulated data did not show a bias on the estimate of *α*, but still the estimated value of *α *was very unstable. Presumably, this difference between real and simulated data is related to the failure of the model to account for the extreme high-expressed tags, which were modeled with a non-parametric component.

When the parameters in the same model were estimated using MCMC, the rate parameter *β *was stable and in agreement with the results from the other methods, but the shape parameter (*α*) was, again, unstable. The estimated parameters for the HTM library N225.111k (a neuroblastoma cell line) are shown in table 2.

### Truncated log-normal-Poisson, Maximum Likelihood

Instead of incorporating a non-parametric component for the high-expressed genes, one may look for a different prior that has a thicker tail. For that purpose, we used a log-normal prior. From a biological point of view, the log-normal distribution is an attractive model for the true gene expression, because it is conventionally used for analysis of microarray data. The disadvantage of that model, however, is that the marginal distribution of the SAGE counts cannot be written on closed form. Therefore, the likelihood must be evaluated with numerical integration.

A simpler method would be to use the method of moments. Since the the probability of a zero count cannot be expressed on closed form, however, numerical integration is still required, though only for the zero counts. Unfortunately, experiments with simulated data showed that the parameters estimated by the method of moments were severely biased (estimated value of *σ*^2 ^typically twice the true value). The ML-algorithm converged in 71 of the 72 libraries, the exception being GSM1130, the library that also gave contradicting *β*-estimates for the truncated and untruncated gamma-Poisson model. Figure 1 shows the frequency plot predicted by the truncated log-normal Poisson model, compared to the empiric frequency plot. Notice that while the model provides a good prediction of the number of strongly expressed tags, it gives a much lower estimate of the number of zero counts than recorded in the data set. That picture is typical.

### Bivariate truncated gamma-Poisson, Maximum Likelihood, HTM data

The fact that the shape parameter in the truncated model could not be estimated on the basis on a single SAGE library is related to the problem of estimating the number of zero counts. In order to solve this problem we fitted the parameters in a bivariate model applied to two libraries. When two libraries are compared, the tags that have positive counts in one library and zero count in the other are reported. If the correlation between the two libraries could be estimated, the number of tags that had zero counts in both libraries could be estimated as well, and we would get a stable estimate of the shape parameter. See the Methods section for details.

We fitted the bivariate model to all pairs from the 18 largest libraries. Of those 153 library pairs, the likelihood maximization algorithm converged in 151 cases. In 127 of those, the four-parameter-model, allowing *α *to be different for the two libraries, provided a significantly better fit (twice log-likelihood ratio higher than the 95-percentile for the chi-square distribution with one degree of freedom). This is not surprising, given that there was considerable dispersion across libraries of the estimated *α *from the untruncated model. With this model, the median of our estimates of the transcriptome size for the subset selected in the HTM was median = 8030 with a quartile range from 6368 to 13767, compared to the 8100 reported in the HTM.

**Figure 2:**
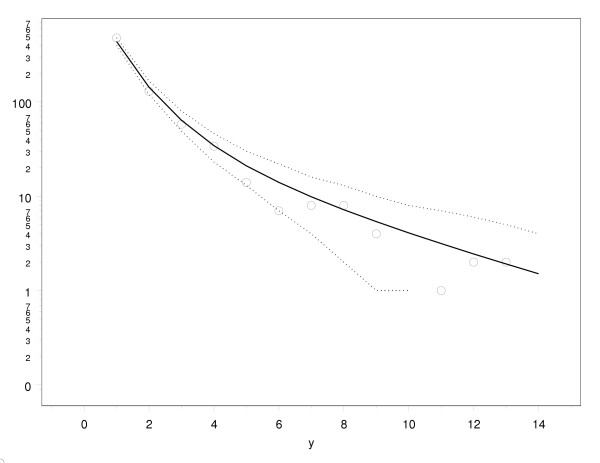
Frequencies of SAGE counts (restricted to genes mapped to a single tag) in IDC-3, given that the same tag had count zero in HTM library 145. Compared to the expected frequencies and 95% confidence bounds from the bivariate model, restricted to tags with counts lower than 15 in both libraries.

**Figure 3:**
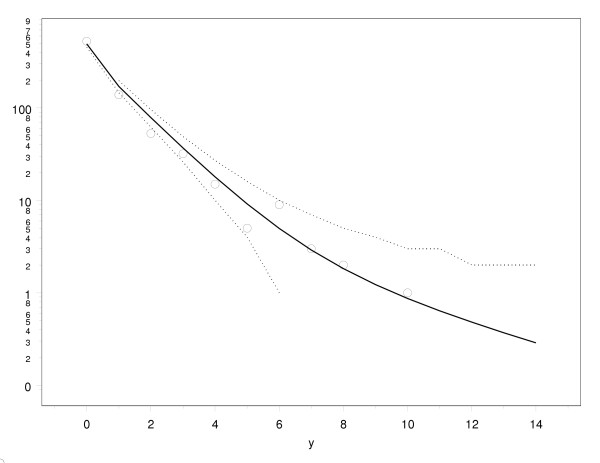
Frequencies of SAGE counts (restricted to genes mapped to a single tag) in IDC-3, given that the same tag had count one in HTM library 145. Compared to the expected frequencies and 95% confidence bounds from the bivariate model, restricted to tags with counts lower than 15 in both libraries.

**Figure 4:**
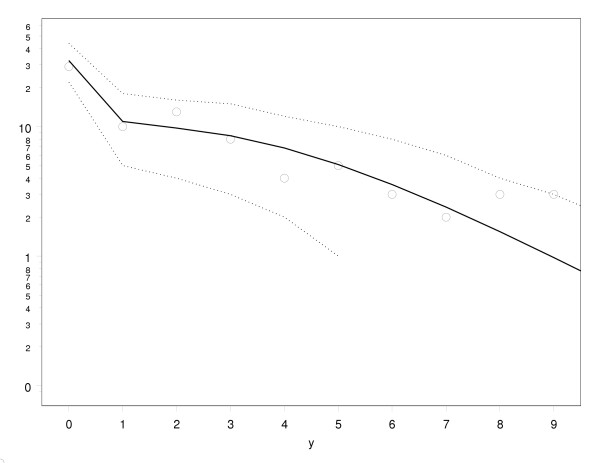
Frequencies of SAGE counts (restricted to genes mapped to a single tag) in IDC-3, given that the same tag had count 5 in HTM library 145. Compared to the expected frequencies and 95% confidence bounds from the bivariate model, restricted to tags with counts lower than 15 in both libraries.

Figures 2, 3 and 4 show how the model compares to the observed data when HTM library IDC-3 (Breast Tumor) is compared to HTM library 145 (Normal brain). Figure 2 shows a frequency plot for IDC-3 for tags that were not observed in libray 145, figure 3 is for tags that had count = 1 in library 145 and figure 4 for tags that had count = 5 in library 145. Notice that the model accurately predicts the number of tags with zero counts (figure 3 and 4), given a positive count in the other library.

**Figure 5:**
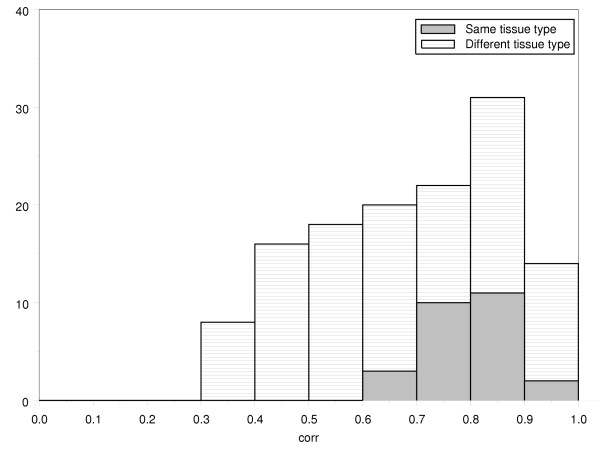
Histograms of the correlation coefficient between two libraries, as estimated with the bivariate model. Library pairs of identical tissue type compared to libraries of different tissue type.

Since the bivariate model specifies the probability of (*Y*_1_, *Y*_2_) = (*y*_1_, *y*_2_) we were able to quantify the correlation between two libraries with the correlation coefficient. As seen in figure 5, a low correlation implies that the two libraries are based on different tissue types, while the converse is not necessarily true.

### Bivariate truncated gamma-Poisson, Maximum Likelihood, raw SAGE libraries

**Figure 6:**
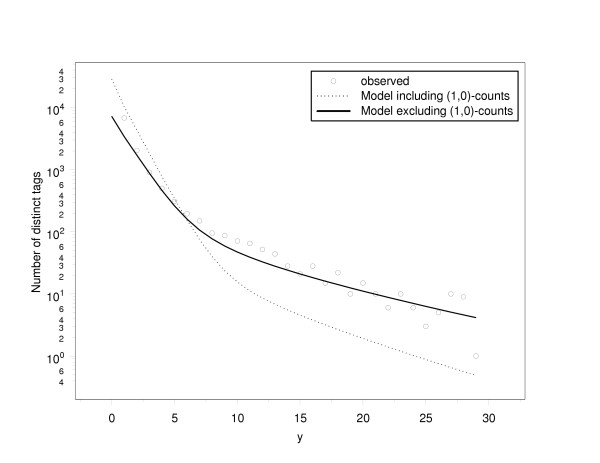
Frequencies of SAGE-genie library 1003, restricted to tags with a count of one in SAGE-genie library 430. Fitted by the bivariate gamma-poisson model with a threshold of 30 for the non-parametric component and allowing for distinct *α*'s in the two libraries (dashed curve). The solid curve shows a fit based on the same model, but discounting tags with a count of one in either library.

As expected, the bivariate model underestimated the number of tags with a count of one, presumably due to sequencing errors. Figure 6 shows a frequency plot for tag counts in SAGE-genie library 1003, conditioned on a count of one in library 430, compared to the predictions from two different versions of the bivariate gamma-poisson-model: the first estimate is based on the assumption that the model fits the data over the entire range, the second is based on the assumption that tags with count one in one library and zero in the other are unreliable and therefore have to be ignored (technically, a non-parametric component accounting for the tags with (1,0) or (0,1) count is assumed). As seen in figure 6, the second model fits much better. The number of tags with a count of one, assigned to the non-parametric component, was 31500 for library 430 and 32100 for library 1003, corresponding to sequencing error rates of 1.0% and 0.7%. This is similar to Beissbart's estimates (between 0.5% and 1.5%).

## Discussion

We have demonstrated that the Poisson distribution with a conjugate gamma prior provides a good fit to real data with the exception of a small population (typically about 1%) of SAGE tags. However, the univariate model does not provide a stable estimate of the shape parameter. For the interpretation of the SAGE counts, this is not so terrible, since the posterior distribution of the true tag expression (*λ*_*t*_) given count *Y*_*t *_is *gamma*(*Y*_*t *_+ *α*, *β *+ 1). With a value of *α *typically between 0 and 0.5, the posterior for distribution of *λ *given a positive count becomes insensitive to *α*. However, the posterior distribution of *λ *given a zero count requires a reliable estimate of *α*.

For the untruncated model, we assumed the total number of expressible tags (in the subset under study) to be 8100 as recorded in the Human Transcriptome Map. One could ask the question whether this set is (roughly) complete or whether there is a significant number of extreme low-expressed tags which have not been recorded in any of the libraries. The fact that the number of expressible tags, as estimated by the bivariate model, had a median of 8030 suggest that the number of tags recorded in the Human Transcriptome Map is of the correct order of magnitude.

The small population of strongly expressed tags has been noted by other authors([16], [4]) before. It is possible that it has a biological or technological interpretation. However, Kuznetsov [7] showed that a discrete Pareto-like distribution accurately predicts the number of high-expressed genes, which suggests that it is a modeling issue rather than a separate group of genes. As seen in figure 1, a Poisson distribution with a log-normal prior, which is also a biologically appealing model, may also be able to predict the number of high-expressed genes. Since the majority of the genes are expressed at such low levels that the difference between the gamma prior and the log-normal prior is small, we decided to base the bivariate model on the gamma prior which is mathematically more convenient.

As a consequence of this choice, the model does not provide a posterior distribution of *λ *given a count above the threshold. Fortunately, this is not so critical because for a high count the posterior mean will be close to the observed count.

Another issue relates to genes mapped to multiple tags. It is reasonable to assume some correlation between two tags representing the same gene. In the human transcriptome map, the counts for those genes were reported as the sum of the tag counts. Suppose a gene is represented by two tags, the count of both being Poisson distributed with intensity *λ *~ *Gamma*(*α*, *β*) and correlation coefficient R. If the total count for the gene is Poisson distributed with intensity λ* ~ *Gamma*(α*, β*), we have

E(λ*)=2E(λ)⇒α*β*=2αβ     (6)
 MathType@MTEF@5@5@+=feaafiart1ev1aaatCvAUfKttLearuWrP9MDH5MBPbIqV92AaeXatLxBI9gBaebbnrfifHhDYfgasaacH8akY=wiFfYdH8Gipec8Eeeu0xXdbba9frFj0=OqFfea0dXdd9vqai=hGuQ8kuc9pgc9s8qqaq=dirpe0xb9q8qiLsFr0=vr0=vr0dc8meaabaqaciaacaGaaeqabaqabeGadaaakeaacqWGfbqrcqGGOaakiiGacqWF7oaBcqGGQaGkcqGGPaqkcqGH9aqpcqaIYaGmcqWGfbqrcqGGOaakcqWF7oaBcqGGPaqkcqGHshI3daWcaaqaaiab=f7aHjabcQcaQaqaaiab=j7aIjabcQcaQaaacqGH9aqpcqaIYaGmdaWcaaqaaiab=f7aHbqaaiab=j7aIbaacaWLjaGaaCzcamaabmaabaGaeGOnaydacaGLOaGaayzkaaaaaa@48D4@

VAR(λ*)=(2+R)VAR(λ)⇒α*β*2=(2+R)αβ2     (7)
 MathType@MTEF@5@5@+=feaafiart1ev1aaatCvAUfKttLearuWrP9MDH5MBPbIqV92AaeXatLxBI9gBaebbnrfifHhDYfgasaacH8akY=wiFfYdH8Gipec8Eeeu0xXdbba9frFj0=OqFfea0dXdd9vqai=hGuQ8kuc9pgc9s8qqaq=dirpe0xb9q8qiLsFr0=vr0=vr0dc8meaabaqaciaacaGaaeqabaqabeGadaaakeaacqWGwbGvcqWGbbqqcqWGsbGucqGGOaakiiGacqWF7oaBcqGGQaGkcqGGPaqkcqGH9aqpcqGGOaakcqaIYaGmcqGHRaWkcqWGsbGucqGGPaqkcqWGwbGvcqWGbbqqcqWGsbGucqGGOaakcqWF7oaBcqGGPaqkcqGHshI3daWcaaqaaiab=f7aHjabcQcaQaqaaiab=j7aIjabcQcaQiabikdaYaaacqGH9aqpcqGGOaakcqaIYaGmcqGHRaWkcqWGsbGucqGGPaqkdaWcaaqaaiab=f7aHbqaaiab=j7aInaaCaaaleqabaGaeGOmaidaaaaakiaaxMaacaWLjaWaaeWaaeaacqaI3aWnaiaawIcacaGLPaaaaaa@5727@

and thus

β*=β1+R     (8)
 MathType@MTEF@5@5@+=feaafiart1ev1aaatCvAUfKttLearuWrP9MDH5MBPbIqV92AaeXatLxBI9gBaebbnrfifHhDYfgasaacH8akY=wiFfYdH8Gipec8Eeeu0xXdbba9frFj0=OqFfea0dXdd9vqai=hGuQ8kuc9pgc9s8qqaq=dirpe0xb9q8qiLsFr0=vr0=vr0dc8meaabaqaciaacaGaaeqabaqabeGadaaakeaaiiGacqWFYoGycqGGQaGkcqGH9aqpdaWcaaqaaiab=j7aIbqaaiabigdaXiabgUcaRiabdkfasbaacaWLjaGaaCzcamaabmaabaGaeGioaGdacaGLOaGaayzkaaaaaa@38AC@

α*=2α1+R     (9)
 MathType@MTEF@5@5@+=feaafiart1ev1aaatCvAUfKttLearuWrP9MDH5MBPbIqV92AaeXatLxBI9gBaebbnrfifHhDYfgasaacH8akY=wiFfYdH8Gipec8Eeeu0xXdbba9frFj0=OqFfea0dXdd9vqai=hGuQ8kuc9pgc9s8qqaq=dirpe0xb9q8qiLsFr0=vr0=vr0dc8meaabaqaciaacaGaaeqabaqabeGadaaakeaaiiGacqWFXoqycqGGQaGkcqGH9aqpdaWcaaqaaiabikdaYiab=f7aHbqaaiabigdaXiabgUcaRiabdkfasbaacaWLjaGaaCzcamaabmaabaGaeGyoaKdacaGLOaGaayzkaaaaaa@399C@

**Figure 7:**
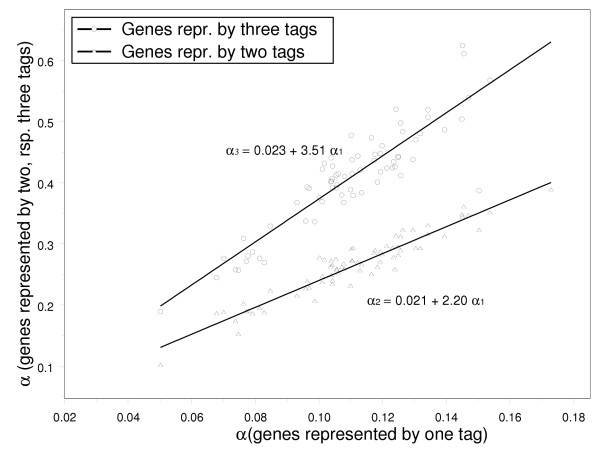
Estimates of *α *in the untruncated model. For each library, the estimates for genes mapped to two tags, and the estimates for genes mapped to three tags, are plotted against the estimate for genesmapped to a single tag.

This shows that if the counts per gene, rather than the counts per tag, are reported in a data set, genes with different numbers of representing tags should be kept separate. As shown in figure 7, the estimated values of *α *for genes mapped to two or three tags are proportional to the estimated values of *α *for the genes mapped to a single tag. This suggests that the tag counts have the same distribution, whether they share the gene with other tags or not. The proportionality constant of 2.2 for genes mapped to two tags and 3.5 for genes mapped to three tags correspond to a correlation coefficient of approximately -0.05. This is a surprising result, since we found positive correlation between tags mapping to the same gene in the data used by Cai [12]. A possible explanation for the negative correlation is that different splicing variants compete for the same transcription product.

By modeling two SAGE libraries with a bivariate truncated negative binomial model, it was possible to achieve a more stable estimate of *α*. More important, the bivariate model has a useful interpretation: the shared gamma process Z is the main effect (gene effect) while the independent gamma process X is the (gene, library) interaction effect. A further generalization of the bivariate model will be to incorporate multiple interaction effects in a multivariate model, for example a third gamma process related to treatment groups.

We have made the distinction between truncated models (only positive tags considered) untruncated model (the set of expressible tags assumed to be known). It could be argued, however, that even when zero counts are reported (such as in multi-library data sets including all tags with a positive count in at least one library), the untruncated models should allow for a an unknown number of non-recorded tags. Such models are called zero-deflated Poisson models.

By the analysis of the HTM data, we have ignored the issue of sequencing errors. Figure 6 suggests that, augmented with a model for the sequencing errors, the model could be applied to raw SAGE data as well.

## Conclusion

SAGE data appear, when sequencing errors are handled properly, to follow a Poisson mixture with a log-normal prior on the Poisson parameter. The gamma prior (leading to a negative binomial distribution for the SAGE counts) provides a good approximation for low counts (up to between 10 and 20, depending mainly on library size). Using a bivariate gamma-Poisson model, the transcriptome size can be estimated from the data; alternatively, the list of expressible tags from the Human Transcriptome Map can be used. Whether one prefers the mathematically convenient gamma prior or a log-normal prior traditionally applied to microarray data, and whether one prefers a parametric or non-parametric model for the high-expressed tags, we believe that the Poisson model is useful for analyzing SAGE data because it separates the sample variance in the Poisson process from the biological variance. Vencio [13] and Cai [12] both demonstrated it to be superior to alternative methods for gene expression differentiation testing and gene clustering, respectively. Assuming a prior distribution of the *λ*'s across the genes could boost their methods further.

## Methods

### Data

We used 72 libraries from the Human Transcriptome Map (HTM) (Caron et al. [17]), which is a compilation of SAGE libraries from different human tissues. It contains expressions of 19825 genes, of which 8100 are represented by a single tag. In this paper, we focus on those genes mainly (we will motivate this choice in the discussion section). The remaining genes were mapped to two or more tags and the reported counts were the sums of the counts of those tags. Unlike raw SAGE libraries, the Human Transcriptome Map includes genes with zero counts, because each library contains counts for all genes that were expressed in at least one library. Also, tag counts considered likely to be false positives (such as sequencing errors) had been removed. See the HTM web-site [18] for details.

We also used two of the short-tag libraries from SAGE-genie [19] (library no. 430 and 1003). Those are raw data, i.e. tags likely to be false positives had not been removed.

### Truncated gamma-Poisson, ML and method of moments

We assumed that for each expression level *i ≥ k *the gamma-poisson-model predicts only a ratio *w*_*i *_≤ 1 of the observed number of tags with that expression level. Therefore, tags with expression level *i *are assigned a weight of *w*_*i *_when estimating *α *and *β*. Initially, the weights were set to 1 and *k *was set to the maximum observed expression level. Now, for each iteration step, *k *was decremented by 1, while *α *and *β *were estimated using the method of moments:

α^=y¯2VAR(y)−y¯2     (10)
 MathType@MTEF@5@5@+=feaafiart1ev1aaatCvAUfKttLearuWrP9MDH5MBPbIqV92AaeXatLxBI9gBaebbnrfifHhDYfgasaacH8akY=wiFfYdH8Gipec8Eeeu0xXdbba9frFj0=OqFfea0dXdd9vqai=hGuQ8kuc9pgc9s8qqaq=dirpe0xb9q8qiLsFr0=vr0=vr0dc8meaabaqaciaacaGaaeqabaqabeGadaaakeaaiiGacuWFXoqygaqcaiabg2da9maalaaabaGafmyEaKNbaebadaahaaWcbeqaaiabikdaYaaaaOqaaiabdAfawjabdgeabjabdkfasjabcIcaOiabdMha5jabcMcaPiabgkHiTiqbdMha5zaaraWaaWbaaSqabeaacqaIYaGmaaaaaOGaaCzcaiaaxMaadaqadaqaaiabigdaXiabicdaWaGaayjkaiaawMcaaaaa@4122@

β^=y¯VAR(y)−y¯2     (11)
 MathType@MTEF@5@5@+=feaafiart1ev1aaatCvAUfKttLearuWrP9MDH5MBPbIqV92AaeXatLxBI9gBaebbnrfifHhDYfgasaacH8akY=wiFfYdH8Gipec8Eeeu0xXdbba9frFj0=OqFfea0dXdd9vqai=hGuQ8kuc9pgc9s8qqaq=dirpe0xb9q8qiLsFr0=vr0=vr0dc8meaabaqaciaacaGaaeqabaqabeGadaaakeaaiiGacuWFYoGygaqcaiabg2da9maalaaabaGafmyEaKNbaebaaeaacqWGwbGvcqWGbbqqcqWGsbGucqGGOaakcqWG5bqEcqGGPaqkcqGHsislcuWG5bqEgaqeamaaCaaaleqabaGaeGOmaidaaaaakiaaxMaacaWLjaWaaeWaaeaacqaIXaqmcqaIXaqmaiaawIcacaGLPaaaaaa@3FFD@

For each *i ≥ k *the expected number of tags *y*_*i*,*exp *_was computed. Now, the weights *w*_*i *_= *y*_*i*,*exp*_/*y*_*i*,*obs *_were computed and used for the estimation of *α *and *β*. This was iterated until Akaike's Information Criteria reached its optimum.

### Truncated gamma-Poisson, Maximum Likelihood and MCMC

The log-likelihood in the truncated negative binomial model, without the non-parametric component for the high-expressed tags, is

∑t(αlog(β)+∑j=0Yt−1log(α+j)−(α+Yt)log(β+1)−log(1−(β1+β)α))     (12)
 MathType@MTEF@5@5@+=feaafiart1ev1aaatCvAUfKttLearuWrP9MDH5MBPbIqV92AaeXatLxBI9gBaebbnrfifHhDYfgasaacH8akY=wiFfYdH8Gipec8Eeeu0xXdbba9frFj0=OqFfea0dXdd9vqai=hGuQ8kuc9pgc9s8qqaq=dirpe0xb9q8qiLsFr0=vr0=vr0dc8meaabaqaciaacaGaaeqabaqabeGadaaakeaadaaeqbqaamaabmaabaacciGae8xSde2exLMBbXgBcf2CPn2qVrwzqf2zLnharyGvLjhzH5wyaGabciaa+XgacaGFVbGaa43zaiabcIcaOiab=j7aIjabcMcaPiabgUcaRmaaqahabaGaa4hBaiaa+9gacaGFNbGaeiikaGIae8xSdeMaey4kaSIaemOAaOMaeiykaKIaeyOeI0IaeiikaGIae8xSdeMaey4kaSIaemywaK1aaSbaaSqaaiabdsha0bqabaGccqGGPaqkcaGFSbGaa43Baiaa+DgacqGGOaakcqWFYoGycqGHRaWkcqaIXaqmcqGGPaqkcqGHsislcaGFSbGaa43Baiaa+DgaaSqaaiabdQgaQjabg2da9iabicdaWaqaaiabdMfaznaaBaaameaacqWG0baDaeqaaSGaeyOeI0IaeGymaedaniabggHiLdGcdaqadaqaaiabigdaXiabgkHiTmaabmaabaWaaSaaaeaacqWFYoGyaeaacqaIXaqmcqGHRaWkcqWFYoGyaaaacaGLOaGaayzkaaWaaWbaaSqabeaacqWFXoqyaaaakiaawIcacaGLPaaaaiaawIcacaGLPaaaaSqaaiabdsha0bqab0GaeyyeIuoakiaaxMaacaWLjaWaaeWaaeaacqaIXaqmcqaIYaGmaiaawIcacaGLPaaaaaa@7BBA@

We augmented this model with a non-parametric component for the high-expressed genes in the same way as the untruncated model described above. We maximized the log-likelihood using a quasi-Newton-Raphson method (S-PLUS function *nlmin*), and made use of the S-PLUS option of computing the gradient and Hessian using the double dogleg step(Venables [20]). As a start guess for the iteration, we used the method of moments (see above). Note that although the sum over all tags is in principle a sum over several thousands indices, in practice it is only a sum over all observed levels of tag expression (counts), due to the discrete nature of the data.

For the threshold k for the non-parametric component, the value found by the method of moments (described above) was used (alternatively k can be handled as another parameter, but it turned out to be difficult to identify). For the prior distribution of the parameters, we used *α *~ exp(1) and *β *~ *gamma*(0.1, 1). In order to quantify the uncertainty on the parameter estimates, we also computed the Bayesian a posteriori distribution of the parameters in the truncated model with non-parametric component using a Markov Chain Monte Carlo (MCMC) algorithm. The MCMC simulations were carried out with WinBugs [21].

### Bivariate Truncated gamma-Poisson, Maximum Likelihood

As a model for the true gene expressions (*λ*) in two libraries (1 and 2), we assumed the trivariate reduction model (Mathal and Moschopoulos [22]):

(*λ*_1_, *λ*_2_) = (*μ*_1 _+ *τ*, *μ*_2 _+ *τ*), *μ*_1_, *μ*_2 _~ *Gamma*(*α*, *β*), *τ *~ *Gamma*(*ρ*, *β*)     (13)

In this model the observed counts *Y*_*t*1_,*Y*_*t*2 _with intensities *λ*_*t*1 _and *λ*_*t*2 _are assumed to arise as sums of a shared component *Z *with intensity *τ *and non-shared components *X*_1 _and *X*_2 _with intensities *μ*_1 _and *μ*_2_.

Assuming that the rate is inversely proportional to the library sizes *n*_1_, *n*_2_, we get

*Y*_1 _= *X*_1 _+*Z*_1_, *Y*_2 _= *X*_2 _+ *Z*_2 _    (14)

*X*_1 _~ *negbinom*(*α*, *β*/*n*_1_), *X*_2 _~ *negbinom*(*α*, *β*/*n*_2_)     (15)

Z1+Z2~negbinom(ρ,βn1+n2)     (16)
 MathType@MTEF@5@5@+=feaafiart1ev1aaatCvAUfKttLearuWrP9MDH5MBPbIqV92AaeXatLxBI9gBaebbnrfifHhDYfgasaacH8akY=wiFfYdH8Gipec8Eeeu0xXdbba9frFj0=OqFfea0dXdd9vqai=hGuQ8kuc9pgc9s8qqaq=dirpe0xb9q8qiLsFr0=vr0=vr0dc8meaabaqaciaacaGaaeqabaqabeGadaaakeaacqWGAbGwdaWgaaWcbaGaeGymaedabeaakiabgUcaRiabdQfaAnaaBaaaleaacqaIYaGmaeqaaOGaeiOFa4NaemOBa4MaemyzauMaem4zaCMaemOyaiMaemyAaKMaemOBa4Maem4Ba8MaemyBa0MaeiikaGccciGae8xWdiNaeiilaWYaaSaaaeaacqWFYoGyaeaacqWGUbGBdaWgaaWcbaGaeGymaedabeaakiabgUcaRiabd6gaUjaaxcW7daWgaaWcbaGaeGOmaidabeaaaaGccqGGPaqkcaWLjaGaaCzcamaabmaabaGaeGymaeJaeGOnaydacaGLOaGaayzkaaaaaa@50FE@

(Z1,Z2)|(Z1+Z2)~binom(Z1+Z2,n1n1+n2)     (17)
 MathType@MTEF@5@5@+=feaafiart1ev1aaatCvAUfKttLearuWrP9MDH5MBPbIqV92AaeXatLxBI9gBaebbnrfifHhDYfgasaacH8akY=wiFfYdH8Gipec8Eeeu0xXdbba9frFj0=OqFfea0dXdd9vqai=hGuQ8kuc9pgc9s8qqaq=dirpe0xb9q8qiLsFr0=vr0=vr0dc8meaabaqaciaacaGaaeqabaqabeGadaaakeaacqGGOaakcqWGAbGwdaWgaaWcbaGaeGymaedabeaakiabcYcaSiabdQfaAnaaBaaaleaacqaIYaGmaeqaaOGaeiykaKIaeiiFaWNaeiikaGIaemOwaO1aaSbaaSqaaiabigdaXaqabaGccqGHRaWkcqWGAbGwdaWgaaWcbaGaeGOmaidabeaakiabcMcaPiabc6ha+jabdkgaIjabdMgaPjabd6gaUjabd+gaVjabd2gaTjabcIcaOiabdQfaAnaaBaaaleaacqaIXaqmaeqaaOGaey4kaSIaemOwaO1aaSbaaSqaaiabikdaYaqabaGccqGGSaaldaWcaaqaaiabd6gaUnaaBaaaleaacqaIXaqmaeqaaaGcbaGaemOBa42aaSbaaSqaaiabigdaXaqabaGccqGHRaWkcqWGUbGBdaWgaaWcbaGaeGOmaidabeaaaaGccqGGPaqkcaWLjaGaaCzcamaabmaabaGaeGymaeJaeG4naCdacaGLOaGaayzkaaaaaa@5AC7@

P((Y1,Y2)=(y1,y2))=∑i=0y1∑j=0y2P((Z1,Z2)=(i,j))P(X1=y1−i)P(X2=y2−i)     (18)
 MathType@MTEF@5@5@+=feaafiart1ev1aaatCvAUfKttLearuWrP9MDH5MBPbIqV92AaeXatLxBI9gBaebbnrfifHhDYfgasaacH8akY=wiFfYdH8Gipec8Eeeu0xXdbba9frFj0=OqFfea0dXdd9vqai=hGuQ8kuc9pgc9s8qqaq=dirpe0xb9q8qiLsFr0=vr0=vr0dc8meaabaqaciaacaGaaeqabaqabeGadaaakeaacqWGqbaudaqadaqaaiabcIcaOiabdMfaznaaBaaaleaacqaIXaqmaeqaaOGaeiilaWIaemywaK1aaSbaaSqaaiabikdaYaqabaGccqGGPaqkcqGH9aqpcqGGOaakcqWG5bqEdaWgaaWcbaGaeGymaedabeaakiabcYcaSiabdMha5naaBaaaleaacqaIYaGmaeqaaOGaeiykaKcacaGLOaGaayzkaaGaeyypa0ZaaabCaeaadaaeWbqaaiabdcfaqnaabmaabaWaaeWaaeaacqWGAbGwdaWgaaWcbaGaeGymaedabeaakiabcYcaSiabdQfaAnaaBaaaleaacqaIYaGmaeqaaaGccaGLOaGaayzkaaGaeyypa0JaeiikaGIaemyAaKMaeiilaWIaemOAaOMaeiykaKcacaGLOaGaayzkaaGaemiuaaLaeiikaGIaemiwaG1aaSbaaSqaaiabigdaXaqabaGccqGH9aqpcqWG5bqEdaWgaaWcbaGaeGymaedabeaakiabgkHiTiabdMgaPjabcMcaPiabdcfaqjabcIcaOiabdIfaynaaBaaaleaacqaIYaGmaeqaaOGaeyypa0JaemyEaK3aaSbaaSqaaiabikdaYaqabaGccqGHsislcqWGPbqAcqGGPaqkaSqaaiabdQgaQjabg2da9iabicdaWaqaaiabdMha5naaBaaameaacqaIYaGmaeqaaaqdcqGHris5aaWcbaGaemyAaKMaeyypa0JaeGimaadabaGaemyEaK3aaSbaaWqaaiabigdaXaqabaaaniabggHiLdGccaWLjaGaaCzcamaabmaabaGaeGymaeJaeGioaGdacaGLOaGaayzkaaaaaa@7C3A@

The correlation between *lambda*_1 _and *lambda*_2 _is

corr(lambda1,lambda2)=VAR(τ)VAR(λ1)VAR(λ2)     (19)
 MathType@MTEF@5@5@+=feaafiart1ev1aaatCvAUfKttLearuWrP9MDH5MBPbIqV92AaeXatLxBI9gBaebbnrfifHhDYfgasaacH8akY=wiFfYdH8Gipec8Eeeu0xXdbba9frFj0=OqFfea0dXdd9vqai=hGuQ8kuc9pgc9s8qqaq=dirpe0xb9q8qiLsFr0=vr0=vr0dc8meaabaqaciaacaGaaeqabaqabeGadaaakeaacqWGJbWycqWGVbWBcqWGYbGCcqWGYbGCcqGGOaakcqWGSbaBcqWGHbqycqWGTbqBcqWGIbGycqWGKbazcqWGHbqydaWgaaWcbaGaeGymaedabeaakiabcYcaSiabdYgaSjabdggaHjabd2gaTjabdkgaIjabdsgaKjabdggaHnaaBaaaleaacqaIYaGmaeqaaOGaeiykaKIaeyypa0ZaaSaaaeaacqWGwbGvcqWGbbqqcqWGsbGucqGGOaakiiGacqWFepaDcqGGPaqkaeaadaGcaaqaaiabdAfawjabdgeabjabdkfasjabcIcaOiab=T7aSnaaBaaaleaacqaIXaqmaeqaaOGaeiykaKIaemOvayLaemyqaeKaemOuaiLaeiikaGIae83UdW2aaSbaaSqaaiabikdaYaqabaGccqGGPaqkaSqabaaaaOGaaCzcaiaaxMaadaqadaqaaiabigdaXiabiMda5aGaayjkaiaawMcaaaaa@63D9@

*VAR*(*λ*.) = *VAR*(*μ*.) + *VAR*(*τ*)     (20)

where the variances of *μ*_1_, *μ*_2 _and *τ *are derived from the gamma distribution. For each pair of libraries from the 19 largest libraries from the Human Transcriptome Map, we estimated the parameters in this model. Anticipating that the model would not fit to the frequencies of counts above the threshold found in the univariate model, which varied between 9 and 22, we restricted the analysis to tags with counts below 15 in both libraries. We also fitted an augmented model in which *X*_1 _and *X*_2 _were allowed to have different values of *α*. In that model, the marginal distributions of *Y*_1 _and *Y*_2 _are negative binomial with parameters (*α*_1 _+ *ρ*, *β*/*n*_1_) and (*α*_2 _+ *ρ*, *β*/*n*_2_), respectively. Note that it is not expected that *α*_1 _is characteristic for library 1 when library 1 is modeled together with different libraries: if library 1 and 2 show a high degree of correlation, *ρ *will be larger at the expense of *α*_1 _and *α*_2_

### Accounting for sequencing errors in raw SAGE data

Raw SAGE data contain a high number of tags with a count of one, many of which, presumably, are artifacts such as sequencing errors. Beissbarth [5] estimated the frequency of such artificial counts to be between 5% and 15% of the total number of tags, corresponding to an error rate of 0.5% to 1.5% per nucleotide. When analyzing the HTM data we assumed that the data had been thoroughly cleaned. But in order to apply our model to raw SAGE data, one needs to account for sequencing errors. We chose to incorporate sequencing errors in the bivariate model by assigning tags with counts one in one library and zero in the other library to a non-parametric component.

## Abbreviations

**HTM **Human Transcriptome Map

**SAGE **Serial Analysis of Gene Expression

**MCMC **Markov Chain Monte Carlo

## Authors' contributions

Both authors were involved in the development of the models. HT implemented the models and performed the computations. Both authors contributed to the manuscript.

## Figures and Tables

**Table 1:** Estimated parameters in the untruncated model. For each of the 72 libraries from the Human Transcriptome Map, the parameters in the untruncated gamma-Poisson model (*α*, *β*) with a non-parametric component for the strongly expressed tags (*y*_*t *_> *thresshold*) were estimated

	*α*	*β*	threshold
Minimum	0.05	0.17	7
1st quartile:	0.10	0.23	10
Median:	0.11	0.27	11
3rd quartile.:	0.12	0.29	13
Maximum:	0.17	1.18	22

**Table 2:** Estimated parameters in the truncated model, library N225.111. In order to assess the uncertainty of the estimated parameters in the truncated gamma-Poisson model with a non-parametric component for the strongly expressed tags, MCMC sampling was carried out on individual libraries. The results from library N225.111 are shown as an example

	*α*	*β*
2.5-percentile(MCMC)	0.0022	0.151
Median(MCMC)	0.047	0.174
97.5-percentile(MCMC)	0.13	0.204
Untruncated model	0.146	0.171
